# 

**Published:** 1878-11-20

**Authors:** 


☞All communications relating to the business of THE RECORD for the years 1877 and 1878, must be addressed DR. R. C. WORD, Managing Editor Southern Medical Record, Atlanta, Ga.

☞Brief and practical communications are solicited on all subjects pertaining to medicine; also reports of cases in practice.

☞Send money by check, postal order or registered letter.

☞Write your name,post-office, county and State plainly.

### SPECIAL NOTICE.

Medical brethren who receive this copy as a sample, are requested to try our Journal for one year, at least, as a matter of experiment.

Those who subscribe now for 1879, may commence with the next or December number, and may remit their subscription in 60 days from date of order.

Any one sending us two new subscribers will be furnished with a copy of "Phyicians Pocket Day Book," worth one dollar. By C. Henri Leonard, M. D. One of the most convenient and beautiful works of the kind ever gotten up.

